# Supplementary material for: The potential for income improvement and biodiversity conservation via specialty coffee in Ethiopia
Source: PeerJ. 2021 Feb 9;9:e10621. doi: 10.7717/peerj.10621 (PMC7879952; doi:10.7717/peerj.10621)
Supplement: Supplemental Information 2 — Survey questions for Yayu primary cooperatives, in English. [file peerj-09-10621-s002.pdf]

Survey Yayu

Q1 Full name and surname ____________________

Q2 Identification number ____________________

Q3 Sex ☐ Male ☐ Female

Q4 Date of birth ☐ Day ☐ Month ☐ Year

Q5 Martital status ☐ Married ☐ Divorced ☐ Single ☐ Widow

Q6 Member of the cooperative since ☐ (Ethiopian Calender Year)

Q7 Highest school grade obtained ☐ No education o ☐ Primary (1-4) ☐ Higher primary (5-8) ☐ High school (9-10) ☐ Preparatory (11-12) ☐ Higher than prepator

Q8 How many people live in your household? (Are you responsible for) ☐

Q9 How many children do you have that live in your household? ☐

Q10 Do all children below 15 go to school? ☐ Yes ☐ No ☐ N/A

Q11 Do one or more members of the household have a cell phone that works? ☐ Yes ☐ No

Q12 Phonenumber ☐

Q13 How many hectares of land do you have? ☐

Q14 How many hectares of coffee do you have? ☐

Q15 Is coffee your most important source of income? ☐ Yes ☐ No

Q16 Does your family work on the farm? ☐ Yes ☐ No

Q17 If yes, which one? ☐ Male ☐ Female ☐ Children under 15?

Q18 Do you use paid daily labour? ☐ Yes ☐ No

Q19 If yes, which one? ☐ Male ☐ Female ☐ Children under 15?

Q20 How many kilo of red cherries did you harvest in 2015/16 ☐ Kilo

Q21 To whom did you sell the red cherries ☐ Cooperative ☐ Kilo ☐ Average price?

☐ Other ☐ Kilo ☐ Average price?

Q22 How many kilo's did you sundry? ☐ Kilo

Q23 How do you dry your cherries? ☐ Drying bed ☐ On the ground ☐ On the tree

Q24 **Surveyor name** ☐ Q25 **Date** ☐ **Day** ☐ **Month** ☐ **Year**

Signature ☐

Signature of farmer ☐
