# Supplementary material for: The potential for income improvement and biodiversity conservation via specialty coffee in Ethiopia
Source: PeerJ. 2021 Feb 9;9:e10621. doi: 10.7717/peerj.10621 (PMC7879952; doi:10.7717/peerj.10621)
Supplement: Supplemental Information 3 — Questionnaire in Amharic (blank form). [file peerj-09-10621-s003.pdf]

Survey Yayu

Q1	maqaa guutuu	______		
Q2	lakkoofsa eenyummeessaa	______		
Q3	saala	☐ dhiira	☐ dhale	
Q4	Bare Dhaiootaa	☐ guyyaa	☐ ji'a	☐ bara
Q5	haala jireenyaa	☐ kan fuudhe	☐ Kan hinfune	☐ kan hike	☐ kan aban manaa irraa du'e
Q6	bara miseensa waldaa hojii gamtaa itti	☐ (Ethiopian Calender Year)		
Q7	sadarkaa barumsaa	☐ kan hin baranne	☐ kutas (1-4)	☐ kutas (5-8)
		☐ kutas (9-10)	☐ kutas (11-12)	☐ isas olii
Q8	baayina maatii	☐		
Q9	Baayina ijoollee wajjin jiraattu	☐		
Q10	Ijorolleen kee umuri 15 gadii hundi ni baretuu?	☐ Eeyyee	☐ Lakki	☐ Hinqabu
Q11	Mobaayilii hojjetu qabdaa	☐ Eeyyee	☐ Hin qabu	
Q12	Yoo eeyyee jette lakkoofsa mobaayilii kee	☐		

Q13	Lafa heektaara meeqa qabda					
Q14	Lafti bunaa heektare meega?					
Q15	Maddaa galii kee keessa inni olaanaan bunaa			Eyyee	Lakki	
Q16	Maatiin kee sii waliin qonna bunaa irratti hojjetuu			Eyyee	Lakki	
Q17	Eyyee, yc o jettee kamiin	Dhiira		Dhalaa		ijollee umuri 15 gadii
Q18	Hojjetaa guyyaa ni fayyadamtaa			Eyyee	Lakki	
Q19	Eyyee, yc o jettee	Dhiira		Dhalaa		ijollee umuri 15 gadii
Q20	Buna diimaa kiiloo meeqa argatte bar 2008				Kilo	
Q21	Buna diimaa argatte eenyutti gurgurte	Waldaa H/Gamtaatti		kiiloo		gatii gidugalessaa
		Daldalaa		kiiloo		gatii gidugaleessaa
Q22	Buna gogaa bara 2008 hangam argatte				Kilo	
Q23	Maal irratti gogsitee buna ka na	Siree buna		Simintuo irratti		muka irratti gugee
Q24	**Maqaa gaafataa**				**Guyyaa**	**guyyaa** / **ji'a** / **bara**
Q25	**Mallattoo gaafataa**					
Q26	**Mallattoo gaafatamaa**					
