# Supplementary material for: The potential for income improvement and biodiversity conservation via specialty coffee in Ethiopia
Source: PeerJ. 2021 Feb 9;9:e10621. doi: 10.7717/peerj.10621 (PMC7879952; doi:10.7717/peerj.10621)
Supplement: Supplemental Information 4 [file peerj-09-10621-s004.pdf]

Survey Yayu

Q1 Full name and surname ______________________________

Q2 Identification number ______________________________

Q3 Sex
☐ Male ☐ Female

Q4 Date of birth
☐ Day ☐ Month ☐ Year

Q5 Martital status ☐ Married ☐ Divorced ☐ Single ☐ Widow

Q6 Member of the cooperative since ☐ (Ethiopian Calender Year)

Q7 Highest school grade obtained
☐ No education o ☐ Primary (1-4) ☐ Higher primary (5-8)
☐ High school (9-10) ☐ Preparatory (11-12) ☐ Higher than prepator

Q8 How many people live in your household?
(Are you responsible for) ☐

Q9 How many children do you have that live in your household? ☐

Q10 Do all children below 15 go to school? ☐ Yes ☐ No ☐ N/A

Q11 Do one or more members of the household have a cell phone that works? ☐ Yes ☐ No

Q12 Phonenumber ☐

Q13 How many hectares of land do you have? [ ]

Q14 How many hectares of coffee do you have? [ ]

Q15 Is coffee your most important source of income? [ ] Yes [ ] No

Q16 Does your family work on the farm? [ ] Yes [ ] No

Q17 If yes, which one? [ ] Male [ ] Female [ ] Children under 15?

Q18 Do you use paid daily labour? [ ] Yes [ ] No

Q19 If yes, which one? [ ] Male [ ] Female [ ] Children under 15?

Q20 How many kilo of red cherries did you harvest in 2015/16 [ ] Kilo

Q21 To whom did you sell the red cherries [ ] Cooperative [ ] Kilo [ ] Average price?

[ ] Other [ ] Kilo [ ] Average price?

Q22 How many kilo's did you sundry? [ ] Kilo

Q23 How do you dry your cherries? [ ] Drying bed [ ] On the ground [ ] On the tree

Q24 How many coffee trees do you have? [ ]

Q25 Do you grow another crop [ ] Yes [ ] No

Q26 Does your coffee has a problem? [ ] Yes [ ] No

Specify

Q27 Do you use chemical fertilizer (agrochemicals) ☐ Yes ☐ No

Q28 Did the cooperative promotion visit your coffee farm

Q29 Age (in years) of your oldest coffee tree ☐ Years

Q30 Age (in years) of your youngest coffee tree ☐ Years

Q31 Do you prune your coffee trees ☐ Yes ☐ No

Q32 Where did you get your seedlings from

Q33 What variety do you grow

Q34 Which date (dd/mm/yy) did your harvest start

Q35 How do you transport the coffee

Q36 **Surveyor name** ☐ Q25 **Date**

**Day**	**Month**	**Year**

Signature ☐

Signature of farmer ☐
